# The Role of Monochromatic Superb Microvascular Index to Predict Malignancy of Solid Focal Lesions: Correlation Between Vascular Index and Histological Bioptic Findings

**DOI:** 10.3390/tomography11040043

**Published:** 2025-04-04

**Authors:** Francesco Giurazza, Luigi Basile, Felice D’Antuono, Fabio Corvino, Antonio Borzelli, Claudio Carrubba, Raffaella Niola

**Affiliations:** 1 Vascular and Interventional Radiology Department, Cardarelli Hospital, Via Antonio Cardarelli 9, 80131 Naples, Italy; felice.dantuono@aocardarelli.it (F.D.); fabio.corvino@aocardarelli.it (F.C.); antonio.borzelli@aocardarelli.it (A.B.); claudio.carrubba@aocardarelli.it (C.C.); raffaella.niola@aocardarelli.it (R.N.); 2 Department of Advanced Biomedical Sciences, University of Naples “Federico II”, 80131 Naples, Italy; basileluigi92@gmail.com

**Keywords:** monochromatic super microvascular index (mSMI), vascular index, ultrasound, biopsy, focal lesions

## Abstract

Objectives: This study aims to assess the potential role of the ultrasound (US) monochromatic Superb Microvascular Index (mSMI) to predict malignancy of solid focal lesions, correlating the vascular index (VI) with bioptic histological results. Methods: In this single-center retrospective analysis, patients undergoing percutaneous US-guided biopsy of solid lesions were considered. Biopsy indication was given by a multidisciplinary team evaluation based on clinical radiological data. Exclusion criteria were: unfeasible SMI evaluations due to poor respiratory compliance, locations not appreciable with the SMI, previous antiangiogenetic chemo/immunotherapies, and inconclusive histological reports. The mSMI examination was conducted in order to visualize extremely low-velocity flows with a high resolution and high frame rate; the VI was semi-automatically calculated. All bioptic procedures were performed under sole US guidance using 16G or 18G needles, immediately after mSMI assessment. Results: Forty-four patients were included (mean age: 64 years; 27 males, 17 females). Liver (15/43), kidneys (9/43), and lymph nodes (6/43) were the most frequent targets. At histopathological analysis, 7 lesions were benign and 37 malignant, metastasis being the most represented. The VI calculated in malignant lesions was statistically higher compared to benign lesions (35.45% and 11% in malignant and benign, respectively; *p*-value 0.013). A threshold VI value of 15.4% was identified to differentiate malignant lesions. The overall diagnostic accuracy of the VI with the mSMI was 0.878, demonstrating a high level of diagnostic accuracy. Conclusions: In this study, the mSMI analysis of solid focal lesions undergoing percutaneous biopsy significantly correlated with histological findings in terms of malignant/benign predictive value, reflecting histological vascular changes in malignant lesions.

## 1. Introduction

Angiogenesis is essential for the growth, invasion, and metastasis of different lesions; previous studies have shown that the flow distribution in malignant lesions is different from benign ones [1,2]. Ultrasound (US) is a key noninvasive diagnostic tool for identifying penetrating vessels and vascular patterns in malignant lesions. Color Doppler flow imaging (CDFI) provides real-time vascular morphology, while power Doppler imaging (PDI) enhances the detection of internal vascularity without being dependent on the US angle [3,4]. However, both CDFI and PDI have limitations in detecting slow flows, restricting their application in evaluating the microvascular flow [5]. Contrast-enhanced ultrasound (CEUS) offers more detailed information on microvascular flow and hemodynamics than CDFI and PDI; however, CEUS requires additional intravenous contrast agent administration and time-consuming post-imaging analysis [6].

Superb microvascular imaging (SMI, Canon Medical Systems, Otawara, Japan) is a modern US technique based on a multidimensional filter to separate flow signals from clutter, preserving slow internal vascularity signals without contrast agents [7]. The SMI offers two imaging modes: color SMI (cSMI) and monochromatic SMI (mSMI), expressed in percentage values. The cSMI simultaneously displays gray-scale and color information, providing a comprehensive overview of vessel continuity and distribution, while the mSMI focuses on flow structure and vessel architecture by cutting the background signal, producing images similar to CEUS without contrast agent administration [8,9]. While the cSMI enhances vessel continuity assessment, the mSMI is specifically designed to improve microvascular flow detection with a monochromatic display, reducing background noise and improving sensitivity to slow-velocity blood flow. Given these advantages, the mSMI was chosen for this study due to its superior ability to depict microvascular structures without contrast agents. Previous studies have extensively documented comparisons between the cSMI and the mSMI, supporting our methodological approach [10,11]. By utilizing the mSMI, we aimed to enhance the visualization of microvascular details that may not be adequately captured by conventional Doppler techniques or even by the cSMI in specific clinical contexts.

Previous studies have shown that the SMI provides higher resolution than CDFI in detecting microvascular flow signals and vascularization in malignant tumors [12]. Further research has demonstrated SMI diagnostic value, especially in hepatic lesions where the SMI provided useful information to differentiate hepatocarcinoma and metastasis from hemangioma [13]. Furthermore, the SMI allows for quantitative analysis through a unique parameter known as the vascular index (VI), which is correlated with tumor microvascular density [14]. Therefore, investigations of the blood supply source and microvascular architecture of malignant lesions using the SMI have the potential to contribute to accurate diagnosis [15]. However, there is a paucity of reports regarding the correlation of the SMI with histological specimens in predicting malignancy values [16].

The aim of this study is now to assess the potential role of the quantitative SMI to predict the malignancy of solid focal lesions, correlating the VI with bioptic histological results.

## 2. Materials and Methods

### 2.1. Sample

This is a single-center retrospective analysis; all patients gave written informed consent to participate in this study. Local ethical committee approval was obtained.

Patients undergoing a percutaneous US-guided biopsy of a solid lesion from January to April 2024 were considered. Biopsy indication was given by a multidisciplinary team evaluation (MDT) based on clinical data and imaging (contrast-enhanced CT and/or MR).

Exclusion criteria were the following: unfeasible SMI evaluations due to poor respiratory compliance, locations not appreciable with SMI, previous antiangiogenetic chemo/immunotherapies, and inconclusive histological reports. Forty-four patients were included in this study, with a mean age of 64 years (range: 17–87 years), 27 males and 17 females.

Liver (15/44), kidneys (9/44), and lymph nodes (6/44) were the most frequent targets (Table 1).

### 2.2. Ultrasound and Superb Microvascular Imaging Examination

Based on a pre-procedural MR or CT scan, the US examination was specifically directed to the biopsy target, immediately before performing the bioptic procedure.

Both US examinations and bioptic procedures were performed by interventional radiologists (>10 years of experience in US-guided procedures), using a modern ultrasound system (Aplio a—Canon^®^ Medical Systems Corporation, Tokyo, Japan).

**Figure 1:**
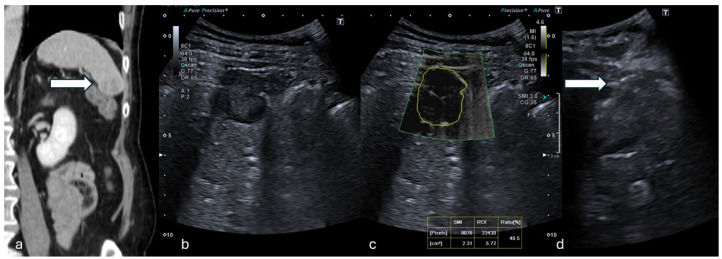
(**a**) Coronal CT reconstruction in venous phase shows a hypodense 16 mm solid nodule of the spleen (white arrow). (**b**) B-mode US showed a dishomogeneous hypoechoic focal lesion with halo sign. (**c**) At mSMI, peripheral and central globular vessels were appreciable, VI measuring 40.5%. (**d**) Percutaneous biopsy using an 18G needle (white arrow). (Histological examination reported a lung cancer metastasis.).

**Figure 2:**
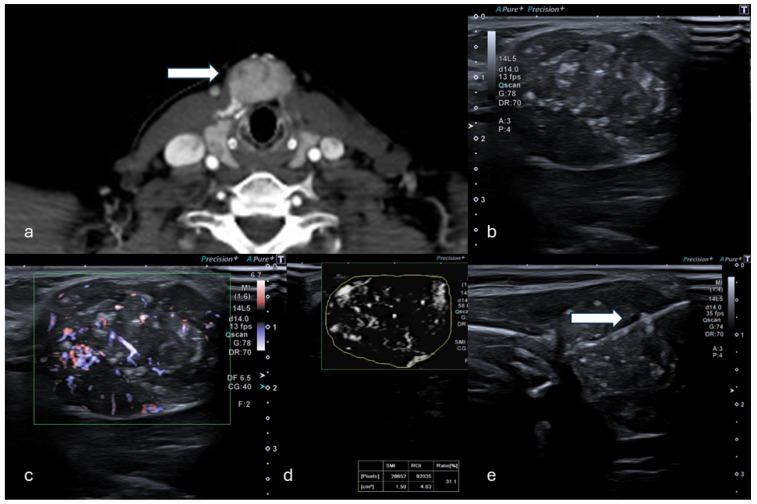
(**a**) Axial CT in venous phase shows a hyperdense, vascularized 24 mm solid lesion of the thyroid (white arrow) in a patient who presented with a palpable mass. (**b**,**c**) B-mode US showed a dishomogeneous hypo-isoechoic lesion. (**d**) mSMI and VI analysis providing a value of 31.1%. (**e**) Percutaneous biopsy using an 18G needle (white arrow). (Histological examination reported a papillary thyroid carcinoma.).

Initially, US images of the lesions were captured with 8C1 convex (Figure 1) and L14-5 linear (Figure 2) transducers, including B-mode, CDFI, and PDI to evaluate tumor characteristics (size, shape, echogenicity, margins, architectural distortion, adjacent vessels) and the proper acoustic window to perform a safe percutaneous approach.

The mSMI examination was conducted asking the patients to breath-hold. US parameters were set to a low-velocity range (1.2–1.6 cm/s) in order to visualize extremely low-velocity flows with high resolution and high frame rates, minimizing flash artifacts (frame rate: 25–30 fps; pulse repetition frequency: 15.4–20.2 kHz; dynamic range of 21 dB). This mode was chosen for its high sensitivity in detecting small- and slow-velocity blood flows. mSMI images were displayed alongside the gray-scale image. Still images and video clips of the mSMI were archived in picture archiving and communication systems.

The VI was semi-automatically calculated directly on the US machine during post-processing imaging evaluation: the lesion boundaries were manually traced by the operator who performed the biopsy on the 2-D mSMI images showing the most representative lesion vascular pattern; the US software furnished the VI expressed in percentage; to minimize bias related to manual tracing, this process was repeated on three consecutive images and the average value was considered.

The total post-processing examination time was approximately 10 min.

### 2.3. Biopsy Technique

Before the biopsy, blood coagulation status was assessed (INR < 1.5 and platelets > 50.000/mL), and patients fasted for 6 h.

All procedures were performed under sole US guidance.

Local anesthetic (10 mL Lidocaine 3%) was administered to the subcutaneous tissues and along the predicted biopsy tract.

Biopsy needles included 16G or 18G Tru-Cut semiautomatic devices (Precisa HS^®^, Rome, Italy). The needle tip was scrubbed with a scalpel to improve its US visibility [17].

Two to four samples were collected from each target. Specimens were routinely fixed in formalin and embedded individually in a paraffin block.

Patients were monitored for 3 h after the procedure and dismissed at home or reconducted to the ward if in an in-hospital setting.

### 2.4. Statistical Analysis

Statistical analysis was performed using SPSS software (v. 21.0, SPSS Inc., Chicago, IL, USA).

Categorical variables were demonstrated as percentages. Descriptive analysis of quantitative variables was expressed as mean value with range (minimum–maximum). The VI was compared among benign and malignant lesions. Mann–Whitney *U* tests were used to compare independent groups. A *p*-value < 0.05 was considered statistically significant.

The diagnostic utility of the VI for estimating malignancy was evaluated using receiver operating characteristic (ROC) curve analysis; the diagnostic accuracy of the VI was provided by calculating the area under the ROC curve. Sensitivity, specificity, positive predictive value (PPV), negative predictive value (NPV), and diagnostic accuracy were provided.

## 3. Results

The histopathological results showed that 7 lesions were benign and 37 lesions were malignant, metastasis being the most represented (Table 2).

The benign lesions included four lymph node lesions, two Warthin’s tumors, and one desmoid tumor. The VI calculated in malignant lesions was statistically higher compared to the VI in benign lesions (median VI: 35.45% and 11.0% in malignant and benign, respectively; *p*-value 0.013).

**Figure 3:**
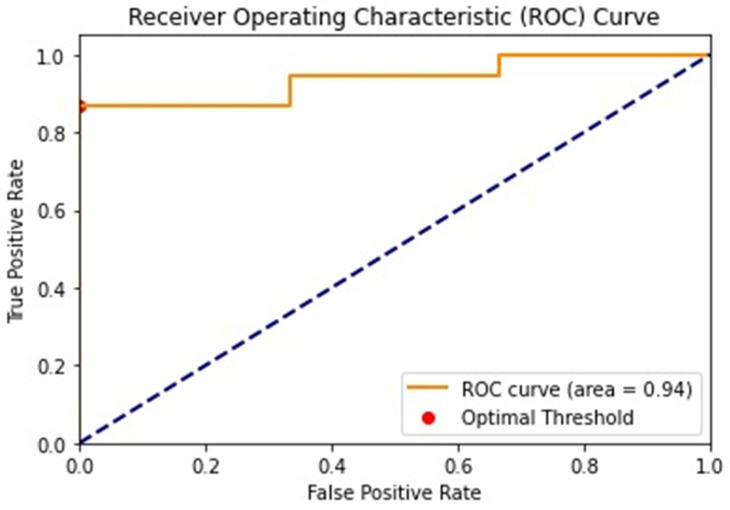
The ROC curve illustrates the diagnostic performance of the mSMI in differentiating benign and malignant lesions. The AUC is 0.94, indicating a high level of accuracy. The optimal threshold value identified for distinguishing malignant lesions is 15.4%, as indicated by the red dots on the curve. ROC: receiver operating characteristic.

The diagnostic performance of the VI by the mSMI in distinguishing between benign and malignant lesions was assessed using the area under the ROC curve (AUC) (Figure 3). The AUC was 0.939, indicating discriminative ability. The 95% confidence interval for the AUC ranged from 0.821 to 1.000, underscoring the reliability of the measure.

A threshold value of 15.4% for the VI was identified for differentiating malignant lesions. At this threshold, the sensitivity was 0.868, and the specificity was 1.0, suggesting high specificity and moderate sensitivity in identifying malignant lesions according to VI examination. The positive predictive value (PPV) was 1.0, indicating that all lesions identified as malignant were true positives. The negative predictive value (NPV) was 0.375, reflecting a lower probability that lesions identified as benign were true negatives.

The overall diagnostic accuracy of the VI by the mSMI was 0.878, demonstrating a high level of diagnostic accuracy.

## 4. Discussion

In this study, mSMI evaluation allowed the identification of the VI values significantly higher in malignant lesions compared to benign; a VI threshold of 15.4 differentiated malignant lesions with a sensitivity of 0.868 and specificity of 1.0.

The SMI enables clinicians to evaluate fine and slow vascular structures without requiring intravenous contrast agents, reducing time, costs, and invasiveness compared to CEUS [18]. Several clinical studies have already highlighted the utility of the SMI in microvascular assessments. Ma et al. analyzed 100 breast lesions and reported that the SMI was sensitive in detecting higher-level blood flow in malignant breast cancers [19]. Similarly, Machado et al. evaluated 50 thyroid lesions and demonstrated that the SMI provided greater blood flow detection and more detailed microvascular architecture than color Doppler flow imaging (CDFI), enhancing diagnostic accuracy [20]. Ayaz et al. evaluated the SMI in assessing ovarian vascularity in children, demonstrating its capability to visualize detailed vascular structures. Their study included 50 pediatric patients, underscoring SMI’s utility in pediatric settings [21]. Mao et al. analyzed the SMI in 75 patients with renal tumors, distinguishing between benign and malignant masses, thereby showcasing its utility in renal pathology [22]. Supporting our results, Kratzer et al. compared the SMI with CEUS in a pilot study of 35 patients with liver metastases, finding that the SMI effectively visualized microvascular structures in malignant liver lesions [23]. Another study evaluating 83 liver lesions found that the SMI was more sensitive than conventional Doppler methods in detecting blood flow in hepatocellular carcinomas (HCCs) [24], paralleling this study’s findings concerning liver metastasis. Further, Kuroda et al. investigated the SMI’s ability to provide a detailed visualization of hepatic vascular architecture in 30 patients with hepatitis C, reinforcing the SMI’s potential in hepatic imaging [25].

Compared to the already existing literature, this study confirms SMI effectiveness in assessing fine microvascularization of solid focal lesions and, furthermore, SMI data with histological findings, providing a comprehensive validation that is often overlooked in the current literature. This correlation strengthens the diagnostic reliability and clinical applicability of the SMI.

Given the SMI’s high sensitivity to microcirculatory changes, immunotherapy and chemotherapy could induce microstructural and microcirculatory alterations during anticancer treatments, reducing SMI reliability during such therapies; so, it is essential to consider these factors when interpreting SMI results in patients undergoing these treatments [26].

This study presents different limitations. First, this study was a single-center retrospective study with a small sample size; a larger-scale prospective clinical study is needed to confirm the proposed findings. Then, the number of benign cases included is low; this is actually expected because biopsies are usually performed on strong malignancy suspicions based on pre-imaging with MR or CT. Finally, SMI assessment has been performed with both linear and convex probes, and this could influence the VI values; similarly, the sample is heterogeneous in terms of histological tissues, and no organ-specific analysis was conducted; however, these aspects allowed us to evaluate the overall predictive value of the SMI in real-life clinical scenarios.

## 5. Conclusions

In this study, mSMI analysis of solid focal lesions undergoing percutaneous biopsy showed a strong correlation with histological findings, in terms of the malignant/benign predictive value. Future research should expand the evaluation of the SMI to a broader spectrum of focal lesions, including those with indeterminate or low malignancy risk, to assess its diagnostic utility beyond high-risk cases. Prospective multicenter studies with larger cohorts are essential to validate these findings and investigate the applicability of the SMI across different organ systems, further establishing its role in non-invasive oncologic diagnostics.

## Figures and Tables

**Table 1:** Lesion locations.

Location	Frequency (%)
liver	15 (34.09%)
kidney	9 (20.45%)
Lymph node	6 (13.64%)
abdominal mass	3 (6.82%)
soft tissues	3 (6.82%)
adrenal glands	2 (4.55%)
salivary glands	2 (4.55%)
spleen	1 (2.27%)
thyroid	1 (2.27%)

**Table 2:** Lesion histology and Median VI values according to benign and malignant origin.

	Benign Lesions	Malignant Lesions
N° of Patients	7	37
Histological results	2 normal lymph nodes 2 Warthin tumors 1 Adrenal adenoma 1 Lymphadenitis 1 Desmoid tumor	18 metastasis 7 clear cell carcinoma 4 lymphomas 3 cholangiocarcinomas 2 gastric adenocarcinomas 1 renal oncocytoma 1 papillary thyroid cancer 1 undifferentiated adenocarcinoma
Median VI values	11	35.45

N°: number; VI: vascular index.

## Data Availability

Study data anonymized are available on demand by contacting the corresponding author.
